# Depression and psychosis related to the absence of visitors and consumption of drugs in male prisoners in Ecuador: a cross sectional study

**DOI:** 10.1186/s12888-019-2227-z

**Published:** 2019-08-07

**Authors:** Andrés Benavides, Juan Chuchuca, David Klaic, William Waters, Miguel Martín, Natalia Romero-Sandoval

**Affiliations:** 1 National Department of Penitentiary Mental Health, National Direction of the First Level of Attention. Ministerio de Salud Pública de Ecuador, Av. Quitumbe Ñan y Av. Amaru Ñan. Plataforma Gubernamental de Desarrollo Social, 170146 Quito, Ecuador; 2 School of Medicine, Universidad Internacional del Ecuador, Av. Jorge Fernández s/n and Simon Bolívar, 170113 Quito, Ecuador; 3 Instituto de Investigación en Salud y Nutrición, Universidad San Francisco de Quito, Diego de Robles y Vía Interoceánica, 170901 Cumbayá, Ecuador; 4 Red GRAAL - Grups de Recerca d’Amèrica i Àfrica Llatines, Barcelona, Spain. Edifici M, Avinguda de Can Domènech, 08193 Bellaterra (Cerdanyola del Vallès), Barcelona Spain; 5 Unidad de Bioestadística, Facultad de Medicina, Universidad Autónoma de Barcelona, Edifici M, Avinguda de Can Domènech, 08193 Bellaterra (Cerdanyola del Vallès), Barcelona Spain

## Abstract

**Background:**

Major mental disorders in prison populations have been recognised as a long-term problem with an important impact on public health. Despite this, screening activities in prisons are still weak in Latin America. We proposed to estimate the prevalence of depression and psychosis and associated factors in the largest Ecuadorian prison for male inmates.

**Methods:**

Cross-sectional study with 309 prisoners chosen at random between January and February 2017, who were administered two structured questionnaires. The first examined socio-demographic and occupational characteristics prior to incarceration and presence of social support networks; the second was the Mini International Neuropsychiatric Interview version in Spanish 5.0.0. We used maximum likelihood tests and multiple logistic regression to assess associations between depression, psychosis and study factors.

**Results:**

The prevalence of depression and psychosis were 50.2% (95% CI 44.6–55.8) and 25.9% (95% CI 21.0–30.8), respectively. The two conditions occurred together in 22.0% of the cases (95% CI 14.8–29.2), and depression or psychosis (D/P) was found in 54.0% (95% CI 48.4–59.6). Consuming drugs while in prison was found in 41.4% (95% CI 35.9–46.9). When age was considered among those consuming drugs, the prevalence of D/P were 72.2, 88.0, and 92.3%, respectively for the age-groups 18–29, 30–39 and 40 years or over (***p***-value < 0.001). Using a multivariate model, we found that not receiving visits (AOR 3.15 95%CI 1.42–6.96) and consuming drugs while in prison (AOR 5.85 95%CI 3.06–11.16) were associated with D/P, while the age effect disappears. Inmates who stopped drug consumption in prison or who had never consumed did not present any significant differences.

**Conclusions:**

Depression and psychosis in prisoners are strongly affected by the absence of visitors and by consumption of drugs in prison. Greater presence of social support networks and preventive actions targeting drug abuse would contribute to improving the mental health of prison inmates.

## Background

Depression and psychotic illness are among the most common mental disorders in prisons [1] and have been recognized as a public health problem for several reasons. Although in prisons, priority is given to treating acute rather than chronic problems, the prevalence of the latter is much higher than in the general population. Prison inmates generally come from socially disadvantaged segments of the community and the presence of mental disorders, along with other communicable and non-communicable diseases, should provoke society’s commitment to justice in order to fight against this inequity [2, 3]. Finally, social isolation, extreme overcrowding, deficient sanitation, lack of health services, inadequate security measures, and violence in prisons can exacerbate or prolong psychiatric symptoms among inmates with mental disorders, even among those with no history of mental illness [4].

A recent systematic review of information from over 24 countries and a population of 33,588 prison inmates found that the prevalence of depression and psychosis was 10.2 and 3.6%, respectively [3]. Data for South America are scarce; however, Andreoli and colleagues (2014) found depression in 12.3% of 1192 male prisoners in the state of Sao Paolo, Brazil between 2006 and 2007 [5] and Pondé and colleagues obtained figures of 17.6, 1.4, and 27.9% for depression, psychosis, and drug abuse, respectively, in 497 prison inmates in the city of Salvador, Brazil, using the Mini International Neuropsychiatric Interview [6].

Since 2009, responsibility for health in prisons has been transferred from ministries of justice to ministries of health in various developed countries [4]. In 2014, this process took place in Ecuador and clinical exams for prisoners became obligatory [7].

The incarceration rate in Ecuador, based on United Nations figures for an estimated national population of 16.89 million in March 2018, was 222 per 100,000 inhabitants [8]. Ecuador thus ranks 60th among the 222 countries for which information is available. In 2011, the average incarceration rate for South American countries was 202 per 100,000 inhabitants, which is one of the highest in the world [9], compared to 145 per 100,000 inhabitants worldwide [3].

In Ecuador, surveillance of diseases related to human behaviour includes alcoholism, dementia, drug dependence, homicide, suicide, and victims of violence, but not depression or psychosis [10]. Consequently, no figures or studies assessing the risk factors are available.

## Methods

### Aims

We proposed to quantify the prevalence of depression or psychosis and relationships with demographic, social, and drug abuse factors among male prison inmates in the Ecuadorian prison with the largest male population, through a cross-sectional study, with the aim of contributing epidemiological information to the mental health system in a penitentiary setting that was recently incorporated into the new health model for prisons.

### Study setting and design

We carried out a cross-sectional study among male inmates aged over 18 years in a prison in the city of Guayaquil, Ecuador. Both the prison and the city are the most densely populated in the country. Prisoners are grouped in areas based on availability of cells rather than by security level or type of crime, although highly dangerous prisoners are held in other prisons.

### Inclusion criteria

Eligible subjects were the 2375 male Ecuadorian or other Spanish-speaking prisoners between 18 and 75 years of age who were held in the prison during the first 3 months of 2017, who had been in the prison for at least 12 months prior to the interview, and whose release was not expected within the next 6 months. Inmates with severe somatic pathologies or security measures preventing the interview were excluded.

### Sample size and sample procedure

Sample size calculations were performed assuming a prevalence of psychosis of 10% [11], requiring a precision of 3% at the 95% confidence level, and a dropout rate of 10%. This yielded a sample size of 362. The percentage of refusal to participate was 14.6%. The reason given for refusal was the unwillingness to participate. The characteristics of the prison and the authorizations to conduct the study made it difficult to complete the sample size with replacement and we obtained a sample of 309 prisoners (84.5%). Participants were selected by simple random selection.

### Methods of data collection

Prisoners were invited to participate in this study in February 2017 in coordination with the Ministry of Justice. They were transferred from their pavilions to the health unit of the prison and those who agreed to participate remained in the doctors’ offices. Each day 20 prisoners were invited. Data were collected by a group of five psychologists with experience in psychological interviewing. The interview was conducted face-to-face with each participant. The purpose of the interview was first explained, and it was made clear that participation was voluntary and that responses would be treated as confidential. Informed consent was obtained before questionnaire administration was begun and privacy was maintained during the interview.

Two structured questionnaires were used. One collected social demographic information as well as visits by family members, place of family residence, and drug abuse history. The second questionnaire was the Spanish version of the Mini International Neuropsychiatric Interview, version 5.0.0 (MINI), screening tool, which was used to assess current status regarding depression, psychosis, and drug abuse (nonalcohol). The latter is defined as consumption in the last month [12] . Depression module is related to Major Depressive Episode and Major Depressive Disorder, and Psychosis module include current Any Psychotic Disorder.

### Operational definitions

Age was grouped into three categories: 18 to 29 years, 30 to 39 years, and 40 years or more. Educational level was categorized as primary school or less and secondary school or higher. Participants were asked about the place of residence of their family (in the city, outside the city, or overseas), whether they had children, whether they had a job prior to incarceration, any past incarceration, and family visits received in the past 3 months. Responses indicating having consumed drugs and current drug abuse according to the MINI were combined to yield three categories: (i) never consumed drugs, (ii) stopped drug consumption during imprisonment, and (iii) consumes drugs in the prison. Depression and psychosis were ascertained using DSM-IV criteria. Finally, we generated a new variable to reflect the presence of at least one of the two mental disorders (D/P), which was used as the dependent variable.

### Statistical analysis

Data were entered with Microsoft Excel 2010 software and analyzed in the Statistical Package for Social Sciences (SPSS, version 21). The variables are described in terms of relative and absolute frequencies. Associations between presence of at least one of the two mental disorders and factors under study were assessed using binary logistic regression. The results are expressed in terms of crude and adjusted OR and their 95% CI. A *p*-value of 0.05 was considered as the cut-off point for statistical significance.

### Ethical considerations

The data were managed by the team of interviewers during the process of data collection, who ensured confidentiality by using an individual alpha-numeric code. Privacy was guaranteed by conducting individual interviews with each participant in a consulting room in the prison’s health unit. The door was closed; a guard waited outside, but this person had no access to any of the information collected during the application of the evaluation instruments.

The printed copies of the completed evaluation instruments were removed from the prison each day. Access to the data was restricted to members of the research team. Under no circumstance was any of the information given to prison personnel or officials of the Ministry of Justice, Ministry of Health, or any other institution. The study was approved by the Human Subject Research Ethics Committee of the Universidad San Francisco de Quito.

## Results

### Demographic and social characteristics

Demographic and social characteristics and data on past incarceration and patterns of drug use are presented in Table 1. Of the 204 prisoners whose families resided in Guayaquil (where the prison is located), 178 (87.3%) stated that they received visits, compared to 80/96 (83.3%) of those whose families resided in another city, and 5/9 (55.6%) of those whose families lived outside the country.

**Table 1:** Description of the sociodemographic factors of the prisoners in the masculine prison, Ecuador

Variable	Categories	Frequency (*n*)	Percentage (%)
Age (years)	18–29	158	51.1
	30–39	77	24.9
	≥ 40	74	23.9
Education	Primary or less	153	49.5
	High school or college	156	50.5
Place of family residence	In the city were the prison is located	204	66.0
	Outside the city were the prison is located	96	31.1
	Out of the country	9	2.9
Children	No	79	25.6
	Yes	230	74.4
Labor situation before imprisonment	Had a job	139	45.0
	Underemployment or unemployment	170	55.0
Previous imprisonments	No	156	50.5
	Yes	153	49.5
Visits	No	46	14.9
	Yes	263	85.1
Drugs	Never	109	35.3
	Stopped using in jail	72	23.3
	Consumes in jail	128	41.4

### Estimation of the prevalence of depression, psychotic illness and drug consumption

Overall, the prevalence of depression was 50.2% (95% CI 44.6–55.8), and of psychosis, 25.9% (95% CI 21.0–30.8); the prevalence of the two disorders simultaneously was 22.0% (95% CI 14.8–29.2), and D/P was present in 54.0% (95% CI 48.4–59.6). The latter condition was found in 95/158 (60.1%) of participants aged 18 to 29 years, in 39/77 (50.6%) of the group aged 30 to 39, and in 33/74 (44.6%) of the group aged 40 or over. Differences were not significant (*p* > 0.05), though. Consuming drugs while in prison was found in 41.4% (95% CI 35.9–46.9) of participants. However, among those who reported drug consumption, the corresponding figures in the three age groups were 65/90 (72.2%), 22/25 (88.0%) and 12/13 (92.3%), respectively, which demonstrates that age interacts with consumption of drugs in the presence of the disorders studied (*p* < 0.001).

### Factors associated with depression or psychotic illness

D/P was found in 86/153 (56.2%) of participants with a primary education or less, in 125/230 (54.3%) of those who had children, and in 95/170 (55.9%) of those who were unemployed prior to incarceration, as well as in 112/204 (54.9%) of those who had family in the same city as the prison, in 51/96 (53.1%) of those who had family outside the city, in 4/9 (44.4%) of those whose families lived overseas.

Table 2 presents associations between depression or psychosis and the factors included in the study. After performing multivariate adjustments, we observed significant adjusted OR values for absence of family visits (AOR 3.15 95%CI 1.42–6.96) and consumption of drugs in prison (AOR 5.85 95%CI 3.06–11.16). Stopping drug consumption upon incarceration and never having consumed drugs behaved similarly. Having a prison history lost its significance after adjusting for the other factors.

**Table 2:** Factors associated with depression or psychosis by bivariable and multivariable logistic regression among masculine prisoners, Ecuador

Variable	Category	n (%)	Crude OR	95%CI	Adjusted OR	95%CI
Visits	No^a^	36 (78.3)				
	Yes	131 (49.8)	3.62	1.73–7.63	3.15	1.42–6.96
Previous imprisonments	No^a^	73 (46.8)				
	Yes	94 (61.4)	1.81	1.15–2.85	1.21	0.72–1.99
Drugs	Never^a^	37 (33.9)				
	Stopped using in jail	31 (43.1)	1.47	0.80–2.71	1.34	0.71–2.55
	Consumes in jail	99 (77.3)	4.51	2.42–8.42	5.85	3.06–11.16
Age (years)	18–29^a^	95 (60.1)				
	30–39	39 (50.6)	0.68	0.39–1.78	1.03	0.54–1.99
	≥ 40	33 (44.6)	0.53	0.03–0.93	1.03	0.51–2.08

^a^Basal category

## Discussion

In penitentiary settings, maintenance and protection of mental health is difficult and availability of resources for diagnosis of mental disorders in prisons is limited [13]. In the present study, we found that one of five participants presented both depression and psychosis, while one of two presented one or the other. The prevalence rate of depression, as well as that of psychosis that we found, seem to be among the highest reported [3].

Some studies conducted in other settings show that rates of common mental disorders in the prison population are double those found in the general population, and quadruple in the case of severe mental disorders [11, 14–16]. Recent studies have reported rates of 8.7 and 20.7% respectively, for depression and psychosis among male prison inmates in Spain [11]; and 6.3 and 12.3%, respectively in Brazil [9]. Similarly, rates of 6.1% for depression were found in Chile [16], and 38. 6% in Colombia [17]. We have not identified any studies published in indexed journals that investigate these topics in Ecuadorian prisons, or in the general population. It has been recognized that some prison inmates present mental disorders prior to incarceration [18], or in other cases that it is in prison where the initial diagnosis is made [19].

We also found a higher prevalence of depression or psychosis among prisoners between 18 and 29 years of age; however, a change was observed in this prevalence upon associating age and consumption of drugs. In this case, we found a very high prevalence of these mental disorders studied among prisoners over 40 years of age who consumed drugs (92.3%). In the logistic regression model, however, the age effect disappeared, not being significant in explaining the variability in the rate of the two mental disorders studied following adjustment for the other factors. Even so, systematic reviews suggest that older prisoners have a higher risk of presenting a single psychiatric disorder [20, 21]. It is known that people with some mental disorder who have committed crimes but who do not receive appropriate treatment, can enter a cycle of re-incidence involving both the mental disorders and criminal acts [22]. Moreover, in low and middle-income countries, prison inmates with mental disorders tend to have such poor access to resources that the risk of suffering abuse and violations of human rights is higher [9].

One systematic review found a prevalence of drug abuse and dependence of 25% among 90,000 prisoners in low and middle-income countries [23] and a figure of 27.1% was found among inmates in prisons in Sao Paulo [5], independently of whether they had mental disorders or not. An updated systematic review and meta-regression analysis in 18,388 prisoners, 64% of whom were male, found prevalences of drug abuse of between 10 and 61% [24]. A recent study in 336 prisoners in Ethiopia (2017) found a prevalence of substance use disorder of 55.9%; unlike our study, this analysis included alcohol abuse and nicotine dependence with an instrument other than the MINI. This study found that lack or poor social support, living in urban areas, psychopathy, and family history of substance use was associated with substance use disorder [25].

We found that among prisoners who consumed drugs in the prison, seven out of ten presented depression or psychosis. We also observed a relationship between not receiving visits and presenting depression or psychosis; 78.3% of prisoners who did not receive visits presented depression or psychosis, and the relationship remained significant after adjusting for the other factors. In Ethiopia, a significant association was found between not having any social support and depression among 727 prisoners [26]. However, Prins (2014) found difficulties in diagnosing these problems in high-income Western countries with sample surveys using interviews and clinical instruments [15].

Mental health service providers and the justice system in prison settings should discuss and agree on operational priorities related to custody, care, security issues and treatment of prison inmates with mental conditions, and should reflect on the system for organizing visits or social support for those who do not receive visits [27]. Falissard and colleagues called for in-depth, collective reflection to answer questions about the diagnosis of mental health in prisons [14]. In particular, it is important to access a user-friendly instrument that can be applied by health technical personnel, who must transfer reliable information to physicians. In this regard, several authors have reiterated the need for early diagnosis and timely treatment of mental disorders in prisons in order to avoid fatal consequences [28–30].

We used the Spanish version of the MINI because of its appropriateness and easy application by psychologists, because it includes the variables of interest in this study (which have been replicated) [31, 32], and because it has been applied to young offenders, for example in Chile [33]. A mental health screening tool should consider important contextual factors in the implementation of screening. Although there are other tests for screening for mental illness [34], they have not been validated in Spanish-speaking countries in Latin America, which is an important limitation to addressing mental health in prisons in the region.

We recognize several limitations of the present study. First, in the selection of participants, we did not consider aspects such as duration of prison sentence, general health status, or time still to be served. These factors could have influenced the mental health test results. Second, the evaluation used in this study did not consider potential differences in prisoners’ mental health on admission to prison, compared with prisoners already incarcerated. A study conducted in Chile found a higher prevalence among those evaluated at the time of admission [16]. Third, the variable of interest (depression or psychosis) was based on a single measurement. An addition to a standardized diagnostic interview could modify the prevalence rates founded. Fourth, prison regulations imposed limitations on the administration of the mental health test and in obtaining our intended sample size: these limitations included the place for the application of the test, the impossibility of contacting prisoner again, and the time authorized for applying the test. Finally, the possibility that drug consumption and not receiving visits might result from mental health problems should be considered.

## Conclusions

Consumption of drugs in prison and the absence of visitors were associated with the mental health of prison inmates. A higher frequency of visits and actions to prevent consumption and abuse of drugs would contribute to improved mental health of prisoners.

## Data Availability

The datasets used and analysed during the current study are available from the corresponding author upon reasonable request.

## Abbreviations

D/P: Depression or Psychosis
MINI: Mini International Neuropsychiatric Interview
